# Effect of Titanium and Molybdenum Cover on the Surface Restructuration of Diamond Single Crystal during Annealing

**DOI:** 10.3390/ma16041650

**Published:** 2023-02-16

**Authors:** Alexander V. Okotrub, Olga V. Sedelnikova, Dmitriy V. Gorodetskiy, Anastasiya D. Fedorenko, Igor P. Asanov, Yury N. Palyanov, Alina V. Lapega, Olga A. Gurova, Lyubov G. Bulusheva

**Affiliations:** 1 Nikolaev Institute of Inorganic Chemistry SB RAS, 630090 Novosibirsk, Russia; 2 Sobolev Institute of Geology and Mineralogy, 630090 Novosibirsk, Russia; 3 Novosibirsk State University, 630090 Novosibirsk, Russia

**Keywords:** diamond, titanium, molybdenum, sp^2^ carbon, annealing, XPS, AFM

## Abstract

Diamond is an important material for electrical and electronic devices. Because the diamond is in contact with the metal in these applications, it becomes necessary to study the metal–diamond interaction and the structure of the interface, in particular, at elevated temperatures. In this work, we study the interaction of the (100) and (111) surfaces of a synthetic diamond single crystal with spattered titanium and molybdenum films. Atomic force microscopy reveals a uniform coating of titanium and the formation of flattened molybdenum nanoparticles. A thin titanium film is completely oxidized upon contact with air and passes from the oxidized state to the carbide state upon annealing in an ultrahigh vacuum at 800 °C. Molybdenum interacts with the (111) diamond surface already at 500 °C, which leads to the carbidization of its nanoparticles and catalytic graphitization of the diamond surface. This process is much slower on the (100) diamond surface; sp^2^-hybridized carbon is formed on the diamond and the top of molybdenum carbide nanoparticles, only when the annealing temperature is raised to 800 °C. The conductivity of the resulting sample is improved when compared to the Ti-coated diamond substrates and the Mo-coated (111) substrate annealed at 800 °C. The presented results could be useful for the development of graphene-on-diamond electronics.

## 1. Introduction

Due to its ultrawide bandgap and high thermal conductivity [1], diamond has attracted the attention of the scientific community already at the silicon sunrise [2]. However, the rarity and high cost of natural stones, as well as the variability of their electronic properties [3], postponed the implementation of diamond electronics until the beginning of the 21st century when methods for the synthesis of centimeter-sized diamond single crystals and their controlled doping appeared [1,4]. Today, diamond technology is developing rapidly. In particular, devices with performances superior to other semiconductors have been reported, including high-frequency and high-power electronics [4,5], detectors [6], and light sources [7].

Graphene is another carbon-based material for electronics applications [8]. In addition to outstanding conductivity and thermal stability, its crystal lattice is commensurate with that of diamond. That provides good adhesion and low contact resistance in the graphene-on-diamond composites [9]. Diamond can be directly transformed into graphite, but this process is associated with a very high activation energy. The graphitization process can occur upon laser irradiation [10,11,12] or high-temperature annealing [9,13,14,15,16]. In the presence of a metal catalyst, the growth of single- and multi-layer graphene on diamond occurs at temperatures substantially lower than those required for the transformation of a bare surface [17,18,19,20,21,22].

Titanium (Ti) and molybdenum (Mo) have good adhesion to the diamond surface and low contact resistance [23,24]. These refractory metals tend to interact strongly with carbon, forming carbides at moderate temperatures. This is widely used to grow a diamond film by chemical vapor deposition (CVD) on Ti and Mo substrates [25,26,27,28,29,30,31]. When high-energy metal atoms are sputtered over diamond [32,33,34] or a coated substrate is annealed [35,36,37,38,39,40], carbon atoms can diffuse through the metal, carbidizing it and promoting the catalytic growth of graphene species. The formation of chemical bonds between diamond, graphene, and metal coating ensures the adhesion of the components and Ohmic contact between them [36,37].

Here, we focused on the transformation of the (100) and (111) surfaces of diamond single crystals coated with very thin layers of titanium and molybdenum under prolonged annealing in high-vacuum conditions of an X-ray photoelectron spectrometer. The samples were annealed at 500 °C and then at 800 °C, and X-ray photoelectron spectroscopy (XPS) measurements were performed after each annealing step to monitor changes in the surface composition. Previous studies of diamonds coated with thick layers of Ti or Mo did not show the formation of carbides as a result of annealing the samples at 500 °C [23,25,35,37,38,40]. However, contamination of the diamond surface can be eliminated by such treatment. The second chosen temperature may be sufficient for the metal carbidization and catalytic transformation of the diamond surface into sp^2^-hybridized species. In addition, this temperature is close to the ceiling temperature for high-power electronics [2]. Thus, this annealing yields an investigation into the interface between the diamond and the contacts at the maximum load. The obtained results could be useful for the development of new graphene-on-diamond technology.

## 2. Materials and Methods

**Figure 1:**
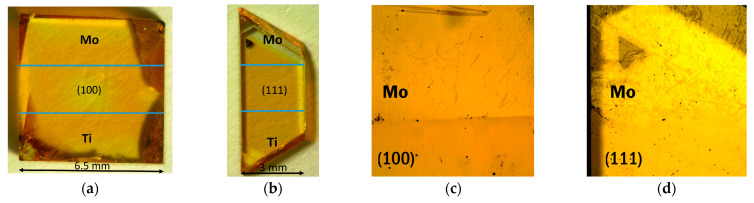
(**a**,**b**) Optical images of (100) and (111) faced diamond substrates with sputtered Mo and Ti layers (the pictures were taken in a transmission mode), (**c**,**d**) images of Mo layers over (100) and (111) faced diamond after 800 °C annealing (the picture was taken in a reflection mode).

Diamond single crystals of lateral sizes from 3 to 7 mm and a height of 1 mm were obtained by the temperature-gradient growth method using a high-pressure apparatus of a “split-sphere” type at a pressure of 5.5 GPa and a temperature of 1400 °C [41]. The starting materials were a graphite rod (99.99% purity), a Ni_0.7_Fe_0.3_ alloy as a solvent catalyst, and synthetic diamonds (ca. 0.5 mm) as seed crystals. Two diamond samples with well-defined (111) and (100) faces were used in this study (Figure 1a,b).

The diamond surfaces were polished and covered with a thin layer of molybdenum or titanium on different sides of the crystals (the top and bottom areas of the crystals shown in Figure 1a,b). To do this, a Mo or Ti target was sputtered for 15 s in a constant power mode (150 W) onto substrates heated to 200 °C using a magnetron sputtering setup (Vacuum System, Novosibirsk, Russia). The treatments were carried out in argon (99.987%) at a pressure of 50 mbar. Areas that should not be covered were masked by aluminum foil. The central regions of both diamond substrates were intact (denoted as D-111 and D-100). The (100) and (111) diamond faces under titanium (molybdenum) layers are denoted as Ti-D-100 and Ti-D-111 (Mo-D-100 and Mo-D-111), respectively.

XPS measurements were performed using a FlexPS spectrometer (Specs, Berlin, Germany), equipped with a hemispherical Phobios-150 analyzer (Specs, Berlin, Germany) and a monochromatic source of Al Kα radiation with an energy of 1486.71 eV. Diamond single crystals were fixed on a sample holder with molybdenum strips and XPS spectra were taken in three different areas, namely from the intact diamond region (central regions of the substrates in Figure 1a,b) and from the diamond surfaces coated with Mo and Ti layers (top and bottom regions of the substrates in Figure 1a,b, respectively). The “small area” mode was used to contract the beam size to 1.4 × 2 mm. After the measurements at room temperature, the samples were annealed in the spectrometer chamber. The sample holder was heated both by a hot filament and an electron beam emitted by the filament at an accelerating voltage of 600 V. The temperature was controlled by a Type K thermocouple sensor (Specs, Berlin, Germany) located on the sample holder. At the heating stage, the pressure in the spectrometer chamber was at least 10^−8^ mbar. The samples were first heated to 500 °C at a heating rate of 3–6 °C min^−1^, then the temperature was fixed for 20 min, followed by cooling the samples to 100 °C for appox. 30–60 min. After measuring the XPS spectra, the samples were heated to 800 °C under the same protocol. XPS spectra measured after annealing of samples at 500 and 800 °C are marked with the prefixes “500” and “800” before the designation (for example, 800-Mo-D-111 indicates the (111) diamond surface coated with Mo and annealed at 800 °C). During the measurements, the pressure in the spectrometer chamber was 10^−9^ mbar. The binding energy was calibrated to the position of the diamond C 1s line (286.0 eV). The atomic concentrations of the elements were calculated from survey spectra taking into account the photoionization cross-sections at a given photon energy. When analyzing high-resolution XPS lines, the background signal was subtracted using Shirley’s method. The lines were fitted using CASA^®^ XPS software. A modified Lorentzian asymmetric lineshape with tail damping was used to approximate the XPS Mo 3d and Ti 2p spectra of the annealed samples. The C 1s lines were normalized to the maximum intensity of each spectrum, and the XPS spectra of titanium and molybdenum measured for each region of coated substrates are given in one-intensity scales.

The structure of the samples annealed at 800 °C was studied by optical microscopy using an Olympus microscope B 52 (Olympus, Tokyo, Japan) in transmission and reflection modes. A more careful investigation was performed by atomic force microscopy (AFM) on a Bruker Dimension Icon atomic force microscope in ScanAsyst mode using standard ScanAsyst-AIR probes (Bruker, Santa Barbara, CA, USA). The cantilever-beam stiffness coefficient was 0.4 N/m, the resonant frequency was 70 kHz, and the nominal rounding radius (AFM sharpness probe) was 2 nm. The scanning field size was 2 µm and the speed was 0.3 Hz at a resolution of 512 × 512 pixels. For a detailed visualization of the morphological features of the studied surfaces, 6 measurements were carried out for each sample, including 2 points in each zone. Raman scattering spectra of the samples annealed at 800 °C were recorded on a LabRAM HR Evolution (Horiba Ltd., Kyoto, Japan) spectrometer at room temperature using an Ar^+^ laser at 514 nm excitation. The spectra were normalized with respect to the diamond peak at 1331 cm^−1^ and are displayed with a slight offset for clarity.

The electrical conductivity of the samples was measured on a Cascade Microtech MPS150 probe station (Cascade Microtech, Beaverton, OR, USA) using a Keithley Source Meter 2401 complex (Tektronix Inc., Beaverton, OR, USA). The measurements were carried out with four probes according to the van der Pauw method. The samples were placed on a dielectric movable table and stainless-steel needle probes were placed on the surface at the maximum possible distance from each other (~1.2 mm). A potential of 21 V was applied to two-side needle probes, and the voltage drop on opposite probes was measured.

## 3. Results

### 3.1. Morphology of Annealed Diamond Surface under Mo and Ti Layers

The (100) and (111) faces of polished single-crystal diamond substrates are pale brown (Figure 1a,b). The sputtering of metal layers does not noticeably change the transparency of the samples. However, the images in the reflection mode show the border between the intact diamond and the diamond covered with Mo after annealing at 800 °C (Figure 1c,d). The difference in optical contrast could be due to the formation of a conducting layer over the Mo coating after heating procedure.

**Figure 2:**
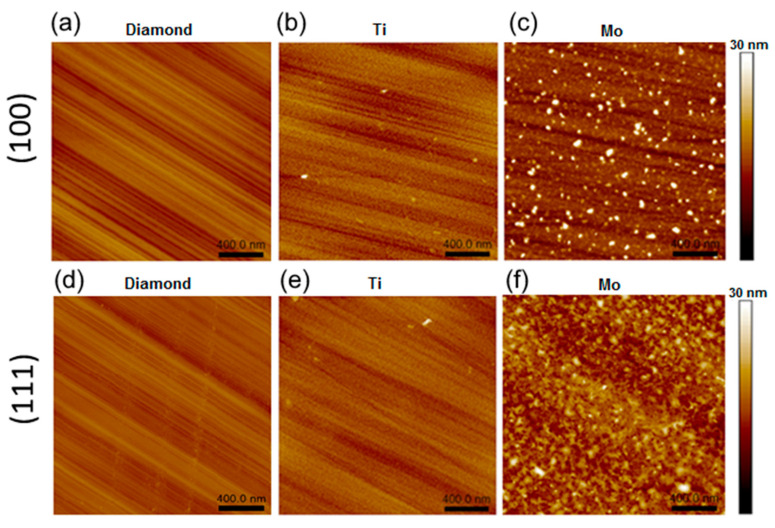
AFM images of 800-D-100 (**a**), 800-Ti-D-100 (**b**), 800-Mo-D-100 (**c**), 800-D-111 (**d**), 800-Ti-D-111 (**e**), and 800-Mo-D-111 (**f**) samples.

AFM images of the samples annealed at 800 °C are shown in Figure 2. The lines on the intact diamond regions are the result of the polishing procedure. For the (100) and (111) faces, the root-mean-square (RMS) roughness S_q_ is about 0.8 and 0.9 nm, respectively (Figure 2a,d). The parameter S_dr_, the so-called development interfacial surface ratio, characterizes the relief and the degree of surface smoothness (for more details, see Supplementary Materials). An S_dr_ value of 0.07% measured for both intact diamond faces means that the surfaces are mirror-polished, and the grooves are flat and skin-deep. Such a grooved surface becomes smoother after the sputtering of a titanium layer followed by annealing. On both faces of the diamond crystals, the titanium coating is a uniform layer consisting of round grains with a lateral size of ca. 15.1 nm and a height of ca. 2.6 nm (see Figure 2b,e and Supplementary Materials for details). The S_q_ values (1.1–1.2 nm) are practically at the level of the uncoated diamond, while the S_dr_ values increase to 3.0–3.5 %. This indicates texturing of the titanium coatings.

**Figure 3:**
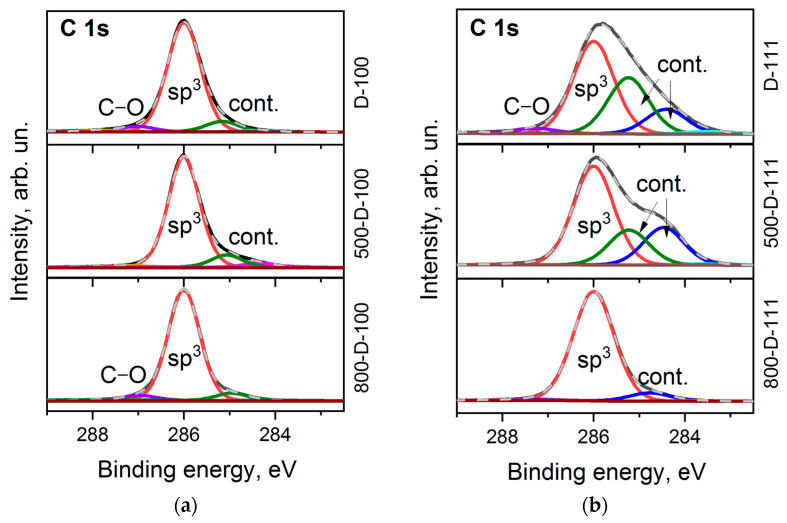
C 1s XPS spectra of D-100 (**a**) and D-111 (**b**) samples before and after annealing at 500 °C and 800 °C.

Samples 800-Mo-D-100 and 800-Mo-D-111 have a morphology, which is quite different from that previously described (Figure 3c,f). For the (100) face, there are flattened grains with a size of 25 nm and a height of 0.7 nm and merged nanoparticles with a diameter of 54.7 nm and a height of 8.2 nm. The maximal height difference of the molybdenum coating is about 24.2 nm, which corresponds to a merged grain. The S_q_ and S_dr_ values are about 2.3 nm and 4.4 %, respectively. The (111) diamond face has a uniform coating, which mainly contains merged grains with an average lateral size of 42 nm and a height of about 4.8 nm. The S_q_ and S_dr_ values for this surface increase to 2.4 nm and to 4.9%, respectively. The higher S_dr_ value for the 800-Mo-D-111 sample indicates the most developed surface compared to other samples.

### 3.2. Surface States of Diamonds

The surface concentrations of elements determined from survey XPS spectra are given in Table 1. Regions D-100 and D-111 consist of carbon (97.2 and 95.7 at%) and oxygen (2.8 and 4.1 at%). Annealing at 800 °C reduces the oxygen content to less than 0.2 at%.

It is commonly accepted that the C 1s line of diamond is a symmetric Gaussian-shaped peak located near 285 eV [42]. However, the shape of the line and its position can vary depending on structural defects [43], polishing [44], and surface functionalization [45]. The C 1s spectra measured for the D-100 and D-111 faces are complex and asymmetric (Figure 3a,b). The best fit was obtained with four components. All spectra are dominated by the bulk sp^3^–carbon peak located at 286.0 eV. The tiny peak that appears at higher binding energies is assigned to oxygen-containing contaminations of the diamond surface [7,46]. There are two peaks shifted by ca. 0.8 and 1.6 eV towards low binding energy. They are attributed to sp^2^-hybridized carbon species, C–H bonds, defects, and band bending [45]. These features are retained in the spectra after the prolonged annealing of the samples at 500 °C. This means high thermal stability of the parent species and strong interaction with the diamond surface. The contribution of supplementary peaks to the D-111 spectrum is more significant (Figure 3b). However, after prolonged annealing at 800 °C, the C 1s spectra of both faces are mainly presented by a symmetric peak at 286.0 eV, which is characteristic of a clean diamond surface [42].

### 3.3. Ti-Coated Diamond Surfaces

The survey XPS spectra of the Ti-coated diamond surfaces show an intense carbon signal and lines from oxygen and titanium (Table 1). The titanium content n_Ti_ is about 6.7 and 4.0 at% for the Ti-D-100 and Ti-D-111 samples, respectively, and almost does not change during annealing (Table 1).

**Figure 4:**
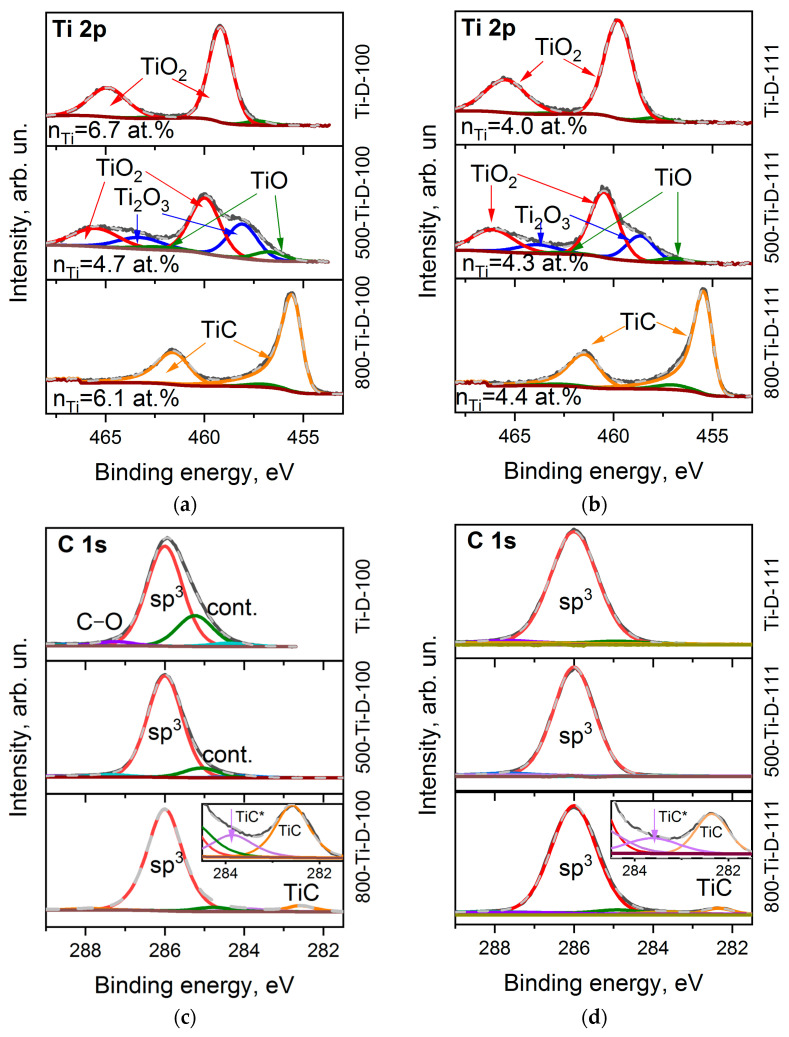
XPS Ti 2p spectra (**a**,b) and C 1s spectra (**c**,**d**) of Ti-D-100 (**a**,**c**) and Ti-D-111 (**b**,**d**) samples before and after annealing at 500 and 800 °C. Insets in (**c**,**d**) zoom the carbide region of C 1s spectra.

Figure 4a,b shows the Ti 2p XPS spectra of the Ti-coated diamond substrates. Before annealing, the spectra consist of two spin-orbit doublets showing dominant components at 459.8 and 465.4 eV assigned to TiO_2_ [47,48] with a small low-energy shoulder. The absence of a signal from metallic titanium (Ti 2p_3/2_ at 453.8 eV [49]) indicates complete oxidation of titanium by air molecules during the synthesis or transfer of the sample from the magnetron system to the XPS spectrometer. The ratio of oxygen (n_O_) to titanium (n_Ti_) is 3.0 and 3.6 for the Ti-D-100 and Ti-D-111 samples, respectively, which exceeds the stoichiometric value for TiO_2_. This means that some of the oxygen atoms are due to functionalized diamond surfaces and the adventitiously deposition of contaminants on the substrates. After annealing the samples at 500 °C, the contribution of lower Ti oxidation state (3+ and 2+) increases, which is revealed as a significant increase in the spectral intensity from the low-energy side of the TiO_2_ states. In particular, the intense components at 458.6 and 463.8 eV and the weak components at 457.0 and 462.6 eV in the spectra of the 500-TiD-100 and 500-Ti-D-111 samples can be attributed to Ti_2_O_3_ and TiO [48,50], respectively. Further annealing removes most of the oxygen from the oxidized titanium layers (the content of titanium oxide states is about 9% and 5% of the total titanium content, respectively). Instead, carbide is formed [51], as we observed from the appearance of asymmetric lines at 455.4 and 461.4 eV.

Despite the presence of noticeable functionalized surface states in the spectra of uncoated diamond substrates, the C 1s XPS spectra of the Ti-D-100 and Ti-D-111 samples are quite symmetric with a centre at 286.0 eV (Figure 4c,d). Apparently, after deposition on the diamond surface, titanium prefers to interact with terminating oxygen rather than being chemically adsorbed on the surface. The presence of physisorbed contaminants between diamond and deposited titanium should lead to the formation of a carbide phase [52]. Because there is no TiC state in the C 1s and Ti 2p spectra of the samples prior to annealing, it can be concluded that these other carbon species were removed from the substrate during pre-heating before sputtering in the magnetron chamber. Annealing in the XPS chamber resulted in further removal of contaminations from the samples and the carbidization of titanium at a temperature of 800 °C. In particular, two carbide states are distinguished. There is a main feature at 282.4–282.6 eV and a minor one at 283.6–283.8 eV. The former state arises dueto the stoichiometric TiC phase, while the latter one can be assigned to the disordered state at the diamond–TiC interface (denoted as TiC* in the insets of Figure 4c,d) [52]. A detailed analysis of the XPS lines showed that about 6 and 4.5 at.% of the carbon surface are involved in the formation of titanium carbide, which is consistent with the n_Ti_ value derived from the survey XPS spectra of the 800-Ti-D-100 and 800-Ti-D-111 samples, respectively.

### 3.4. Mo-Coated Diamond Surfaces

The XPS survey spectra of the Mo-coated diamond surfaces indicate the presence of carbon, molybdenum, and oxygen. The proportion of Mo:O is about 0.6, and the contribution of carbon to the overall spectra decreases to 49.8 and 75.5 at.%, relative to the value for the intact (100) and (111) diamond faces due to the shielding effect from the metal layers.

**Figure 5:**
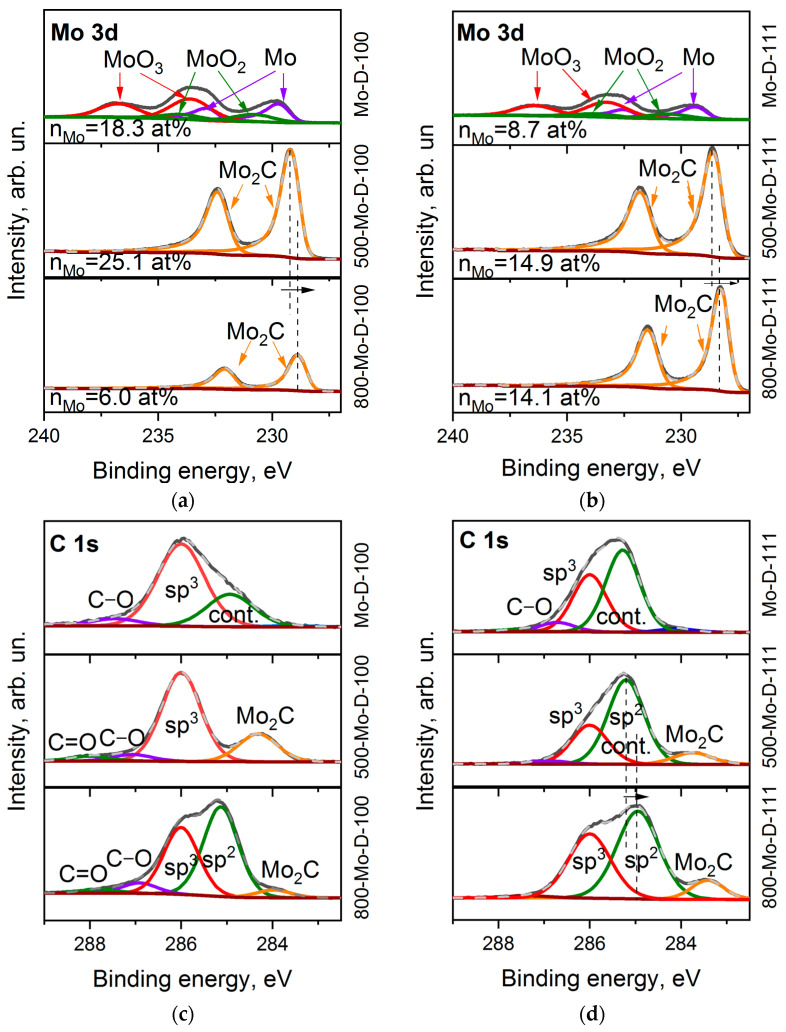
XPS Mo 3d spectra (**a**,**b**) and C 1s spectra (**c**,**d**) of Mo-D-100 (**a**,**c**) and Mo-D-111 (**b**,**d**) samples before and after annealing at 500 and 800 °C.

Six components in the Mo 3d spectrum of Mo-D-100 (Figure 5a) refer to MoO_3_ (at 233.7 and 236.4 eV, 48.8%,) [53,54], MoO_2_ species (at 230.6 and 233.2 eV, 16.9%) [54,55], and molybdenum in the metallic phase (at 229.5 and 232.4 eV, 34.3%) [55]. The ratio of these species in the Mo-D-111 sample is 51.0: 15.6:33.4 (Figure 5b). After annealing at 500 °C, oxygen is mainly removed from the sample and the molybdenum content almost doubles (Table 1). This indicates the removal of adventitious deposits from the surface. For annealed samples, only the Mo_2_C doublet is presented [56]. With an increase in the treatment temperature to 800 °C, the carbide peaks shift by 0.3 eV towards a lower binding energy. One can assume that the transformation of carbide from an amorphous state to a hexagonal phase occurs at high temperatures [34]. In addition, we note that the content of molybdenum in samples 500-Mo-D-111 and 800-Mo-D-111 is the same, while the n_Mo_ value decreases significantly for the Mo-coated (100) diamond surface with increasing annealing temperature. This allows us to assume that the morphology of the 800-Mo-D-100 sample is significantly different from the rest.

The C 1s XPS spectra of the Mo-coated diamond surfaces were fitted by three components (Figure 5c,d). The peak at 286.0 eV corresponds to sp^3^–carbon, a small component at 286.7–287.4 eV is associated with carbon bonded to oxygen, and the peak at 285.0–285.2 eV is presumably a superposition of adventitious carbon deposited above the molybdenum layer and the states of the diamond surface (see Figure 3). When molybdenum is sputtered, the intensity of the contamination peak increases relative to its value in the uncoated diamond substrate. This effect can be explained by a decrease in the escape depth of photoelectrons from coated diamonds, which leads to a thinning of the probed region of sp^3^–hybridized carbon species. The spectrum of the 500-Mo-D-100 sample shows a symmetric diamond peak at 286.0 eV, supplemented by a low-energy molybdenum carbide component at 284.2 eV and oxygen-containing groups. As the temperature increases to 800 °C, a new high-intensity peak appears at 285.2 eV. This feature could be assigned to the formation of graphene-like fragments. The C 1s XPS spectra of the Mo-D-111 sample annealed at 500 and 800 °C are very similar to that obtained for the 800-Mo-D-100 sample, demonstrating the components of sp^3^, sp^2^ carbons, and carbide. It can be assumed that the formation of sp^2^-hybridized carbon in this sample begins at a temperature below 500 °C.

**Figure 6:**
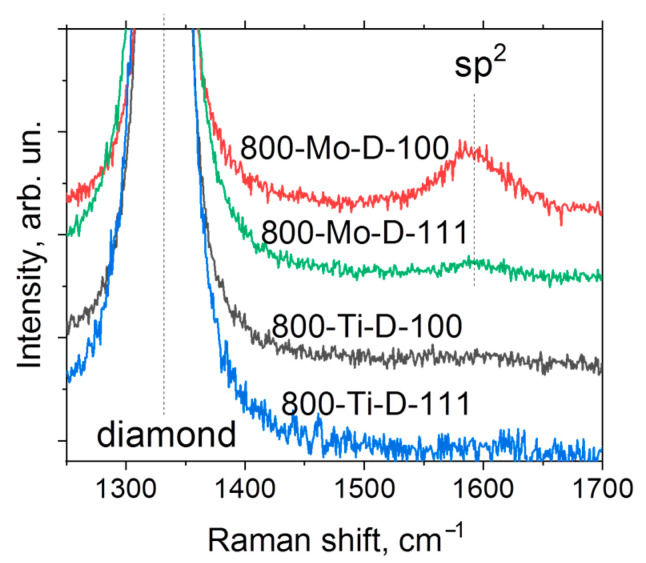
Raman spectra of metal-coated diamond substrates annealed at 800 °C.

Figure 6 shows the Raman spectra of the metal-coated samples annealed at 800 °C. There is a characteristic peak of diamond located at 1331 cm^−1^. Additionally, the spectra of Mo-coated substrates annealed at 800 °C contain a low-intensity broad peak centered at 1592 cm^−1^, which indicates the presence of sp^2^-hybridized carbon in agreement with the above XPS results. The difference between the intensities of this broad peak in the 800-Mo-D-111 and 800-Mo-D-100 spectra is related to the features of the distribution of sp^2^-hybridized carbon over the surfaces. More details are described below.

## 4. Discussion

**Figure 7:**
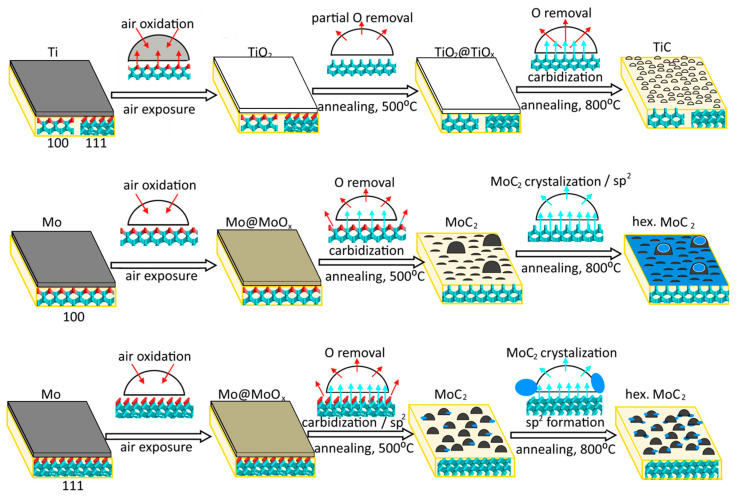
Transformation of titanium and molybdenum coatings deposited on diamond (100) and (111) surfaces under annealing. Cyan and red balls show carbon and oxygen atoms. Cyan and red arrows illustrate carbon diffusion into deposited nanoparticles and oxygen removal during annealing, respectively. Blue particles represent sp^2^ carbon species.

The above results show that the morphology of the coating layer depends on the metal type, the diamond face, and the annealing temperature (Figure 7). Unfortunately, we did not study our samples by AFM before heating. However, we believe that a metal film deposited on the diamond plate at low temperatures should have a fairly uniform and continuous structure.

The film thickness is about 1–2 monolayers of titanium and 3–5 monolayers of molybdenum, as estimated from the XPS data using the Thickogram method [57] (see Supplementary Materials for details). The thicker molybdenum layer, compared to the titanium obtained with the same magnetron settings, can be associated with the increasing role of the scattering of lighter atoms by the gaseous medium. In air, the deposited titanium film is fully oxidized to TiO_2_, while the surface oxidation of the molybdenum film occurs with the preservation of metallic core. This is caused by the greater thickness of the molybdenum film. The C 1s spectra of Ti-coated diamond substrates are similar to the spectrum of diamond annealed at 800 °C, suggesting that Ti readily reacts with the diamond surface, which is consistent with recently published results [36,58]. Therefore, the titanium film could act as a cleaning agent to purify the surface of the diamond. The deposition of molybdenum does not affect diamond, which indicates the physisorption character of interface interactions in this case.

Prolonged annealing removes contaminations deposited on the diamond surface during transfer from the magnetron system to the XPS spectrometer. Simultaneously, metal oxides are reduced to metals that react with carbon to form TiC after annealing Ti-coated diamond at 800 °C and Mo_2_C after annealing Mo-coated diamond at 500 °C. According to the AFM images, the metal films turn into nanoparticles after all heating steps. The almost constant value of n_Ti_ before and after annealing (Table 1) indicates that XPS senses the same amount of Ti throughout the study. This agrees with the close values of the AFM-measured height of TiC nanoparticles (2.6 nm) and the attenuation length of photoelectrons (ca. 2 nm [59]), which indicates that the entire volume of carbide nanoparticles forms an XPS signal. 

Contrary to the above results, the n_Mo_ value increases after annealing the Mo-coated diamond at 500 °C (Table 1). This is due to the removal of contaminations that remain on the diamond surface after molybdenum sputtering. Further heating of the Mo-D-111 sample does not affect the molybdenum content, implying that no significant change in the geometry of carbide component occurs. This suggests that the transformation of the molybdenum coating into nanoparticles takes place already at 500 °C. A sharp decrease in n_Mo_ from 25.1 at.% for the 500-Mo-D-100 sample to 6.0 at.% for the 800-Mo-D-100 sample can be explained by the formation of nanoparticles, whose height (8.2 nm) is significantly larger than the value of the attenuation photoelectron length (ca. 2.3 nm [59]). This means that the XPS Mo 3d spectrum of 800-Mo-D-100 sample reflects only the outer shell of these huge particles, ignoring their volume.

The above discussion leads us to the conclusion that the transformation of the metal layer into nanoparticles on the diamond surface is associated with the formation of carbides, which occurs near 500 and 800 °C for Mo- and Ti-coated substrates, respectively.

The DC measurement reveals a resistivity of about 4 kOhm/□ for the 800-Mo-D-100 sample, while other areas of diamond substrates show a dielectric character. The appearance of conductivity could be associated with the formation of sp^2^ carbon, which was detected in the XPS and Raman spectra of the annealed Mo-coated diamond substrates. Two processes can be responsible for this, namely the catalytic graphitization of the diamond surface and the formation of a graphitic coating over carbide nanoparticles. The melting of a molybdenum film, followed by its disintegration, could produce very small Mo nanoparticles, as was shown in our previous work devoted to the graphitization of iron-coated diamond [22]. These particles immerse into the diamond, catalyzing the transformation of sp^3^ carbon to sp^2^ carbon. We assume that such catalytic graphitization could take place near 800 and 500 °C for Mo-coated (100) and (111) diamond surfaces, respectively. The lower temperature required for the graphitization of the Mo-D-111 sample correlates with the theoretical results, which showed preferential exfoliation of the (111) diamond face [15,16]. Additionally, graphitic layers can be formed on top of molybdenum carbide nanoparticles, as shown earlier [60]. This process, along with the process of the disintegration of the metal film, could also be the reason for the decrease in n_Mo_ value for the Mo-coated (100) face of diamond after annealing at 800 °C (Table 1). If we assume that 800-Mo-D-100 consists of a diamond surface under the Mo_2_C nanoparticles, which are coated with a graphitic mantle, then the thickness of the sp^2^ carbon species is about 2.9 nm, as estimated from the intensity of the Mo 3d lines (see Supplementary Material for details). Because the bulk solubility of carbon in Mo_2_C is a decisive factor for separating graphite-like coating from molybdenum carbide [60], the presence of the biggest carbide nanoparticles in the 800-Mo-D-100 sample could be critical to launching this process. The simultaneous formation of sp^2^ species at the diamond (100) surface and above the carbide nanoparticles increases their contribution to the XPS and Raman spectra and enhances the electronic transport through other samples.

## 5. Conclusions

The transformation of diamond coated by thin films of titanium and molybdenum was studied at high temperatures. For this purpose, two millimeter-sized diamond single-crystals with (100) and (111) faces were coated with metals and annealed at 500 and 800 °C under high-vacuum conditions. The composition of the diamond and coatings was determined from XPS spectra recorded after each treatment without exposing the samples to air. Moreover, the morphology and conductivity of coated diamond surfaces annealed at 800 °C were studied. The presented results show the potential of annealed diamond with refractory metals coating in electronic devices. The transformation of the metal-on-diamond layer to carbides nanoparticles at elevated temperatures (500 °C for Mo and 800 °C for Ti) promises the formation of stable contact between the components. The formation of sp^2^ carbon fragments for Mo-coated diamonds after annealing improves the conduction properties of the substrates. This is important for creating high-frequency transistors, radiation detectors, and transparent contacts, and can be used to develop graphene-on-diamond technology.

## Figures and Tables

**Table 1:** XPS-derived concentration of carbon, oxygen, titanium, and molybdenum in the different regions of diamond substrates before and after annealing at 500 and 800 °C.

Sample	Element Concentration, at.%				Sample	Element Concentration, at.%			
	n_C_	n_O_	n_Ti_	n_Mo_		n_C_	n_O_	n_Ti_	n_Mo_
D-100	97.2	2.8	-	-	D-111	95.7	4.1	0.2	-
500-D-100	99.8	0.2	-	-	500-D-111	99.3	0.6	-	0.1
800-D-100	100.0	0.0	-	-	800-D-111	99.7	0.2	-	0.1
Ti-D-100	74.3	20.0	6.7	-	Ti-D-111	81.8	14.2	4.0	-
500-Ti-D-100	84.9	10.4	4.7	-	500-Ti-D-111	85.9	9.8	4.3	-
800-Ti-D-100	93.7	0.3	6.1	-	800-Ti-D-111	94.8	0.7	4.4	-
Mo-D-100	49.8	31.3	-	18.3	Mo-D-111	75.7	15.6	-	8.7
500-Mo-D-100	73.7	1.2	-	25.1	500-Mo-D-111	82.4	2.7	-	14.9
800-Mo-D-100	93.9	0.1	-	6.0	800-Mo-D-111	85.9	0.0	-	14.1

## Data Availability

The data presented in this study are available on request from the corresponding author.

## Supplementary Materials

The following supporting information can be downloaded at: https://www.mdpi.com/article/10.3390/ma16041650/s1, Figure S1: AFM images of metal-coated diamond substrates annealed at 800 °C; Figure S2: Height profiles through the lines indicated in Figure S1; Figure S3: Raman spectra of 800-Ti-D-100, 800-Ti-D-111, 800-Mo-D-100, and 800-Mo-D-111 samples normalized at the noise level; Table S1: Average height and size of nanoparticles, the root-mean-square roughness (S_q_), and the surface development (S_dr_) in the annealed samples determined across the profile lines shown in Figures S1 and S2; details of estimation of thicknesses of layers of sputtered metals and sp^2^ carbon from XPS data. References [57,61] are cited in the supplementary materials.
