# Supplementary material for: Effect of Titanium and Molybdenum Cover on the Surface Restructuration of Diamond Single Crystal during Annealing
Source: Materials (Basel). 2023 Feb 16;16(4):1650. doi: 10.3390/ma16041650 (PMC9965767; doi:10.3390/ma16041650)
Supplement: Supplementary file 1 [file materials-16-01650-s001.zip › materials-2211020-supplementary.pdf]

Effect of Titanium and Molybdenum Cover on the Surface Restructuration of Diamond Single Crystal during Annealing

Alexander V. Okotrub [1,*], Olga V. Sedelnikova [1,*], Dmitriy V. Gorodetskiy [1], Anastasiya D. Fedorenko [1], Igor P. Asanov [1], Yury N. Palyanov [2], Alina V. Lapega [3], Olga A. Gurova [1] and Lyubov G. Bulusheva [1]

[1] Nikolaev Institute of Inorganic Chemistry SB RAS, 630090 Novosibirsk, Russia
[2] Sobolev Institute of Geology and Mineralogy, 630090 Novosibirsk, Russia
[3] Novosibirsk State University, 630090 Novosibirsk, Russia
* Correspondence: spectrum@niic.nsc.ru (A.V.O.); o.sedelnikova@gmail.com (O.V.S.)

1. Morphology of titanium and molybdenum coatings after annealing at 800 °C

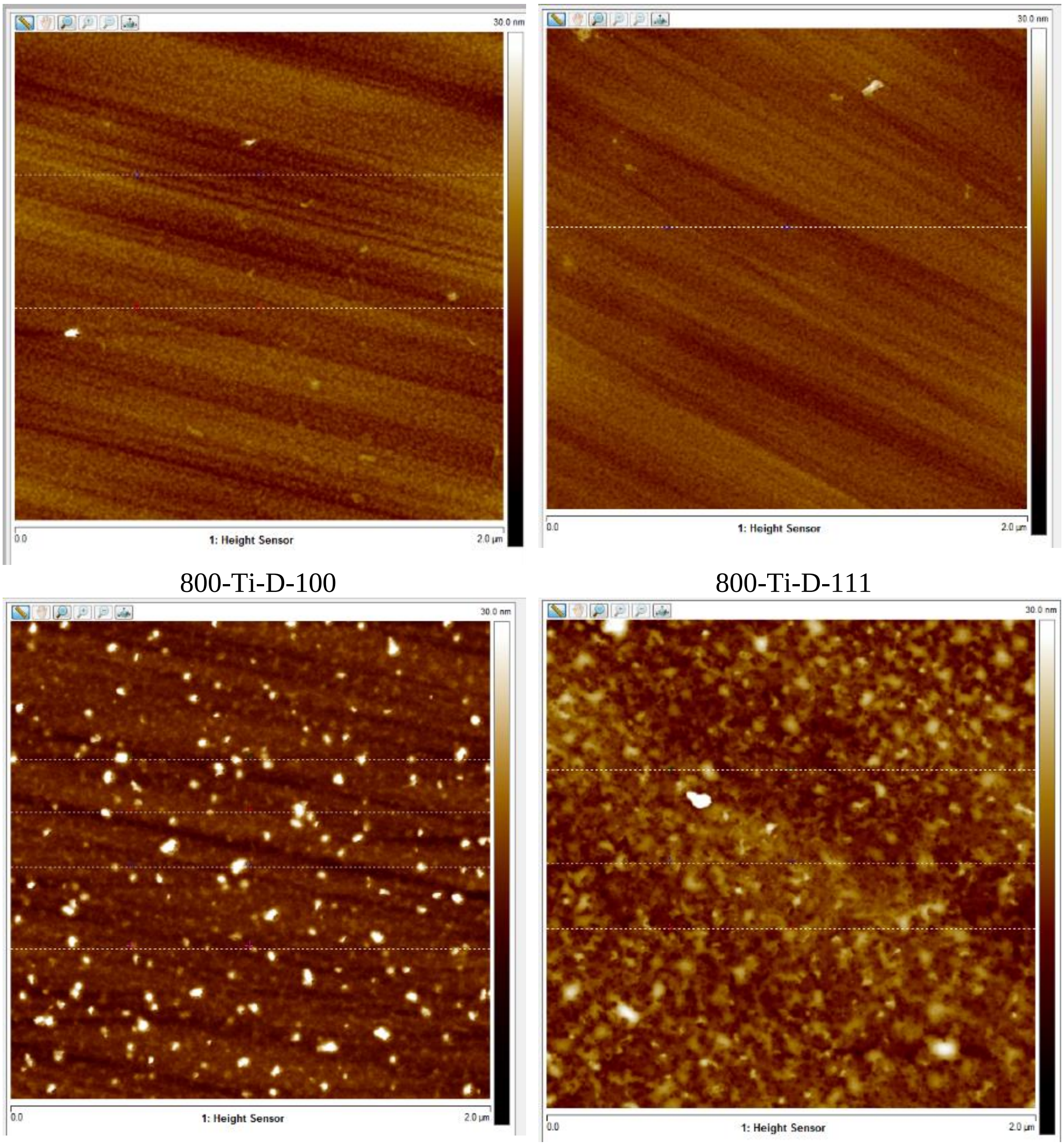

Figure S1. AFM images of metal-coated diamond substrates annealed at 800 °C. Dotted lines used for height profile plots (see Figure S2).

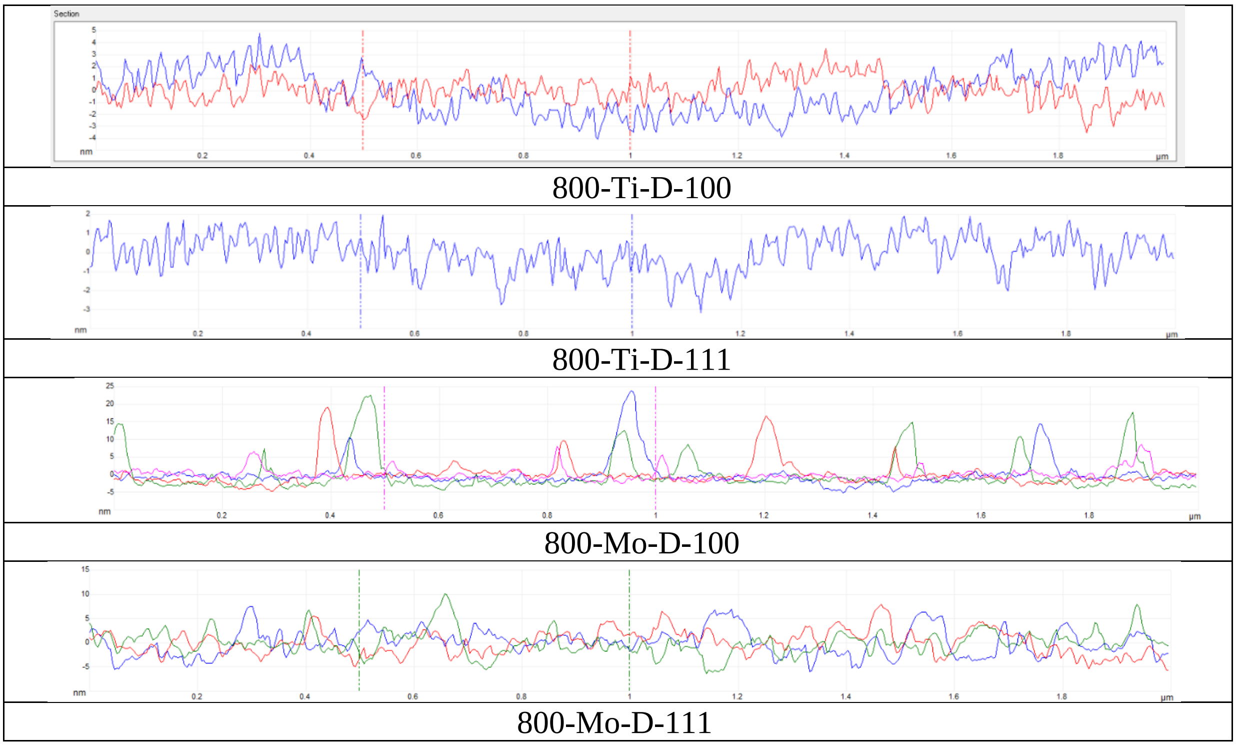

Figure S2. Height profiles through the lines indicated in Figure S1.

Table S1. Average height and size of nanoparticles, the root mean square roughness (S_q), and the development interfacial surface ratio (S_{dr}) in the annealed samples determined across the profile lines shown in Figures S1 and S2.

	Ti		Mo	
	800-Ti-D-100	800-Ti-D-111	800-M0-D-100	800-Mo-D-111
Height, nm	2.74	2.40	0.69 / 8.23	4.80
Size, nm	14.08	16.19	23.0 / 54.74	41.97
S_q, nm	1.05	1.24	2.33	2.44
S_{dr}, %	3.48	3.04	4.35	4.85

The parameter S_{dr}, the so-called development interfacial surface ratio, characterizes the relief and the degree of surface smoothness. S_{dr} is the ratio between the real surface area (Ar) and the geometrical area (A) using the following equation: $S_{dr} = \frac{1}{A}[\iint_A (\sqrt{\left[1 + (\frac{\partial z(x,y)}{\partial x})^2 + (\frac{\partial z(x,y)}{\partial y})^2\right]} - 1)dxdy]$, where A is equal to the scan size.

3. Raman spectra

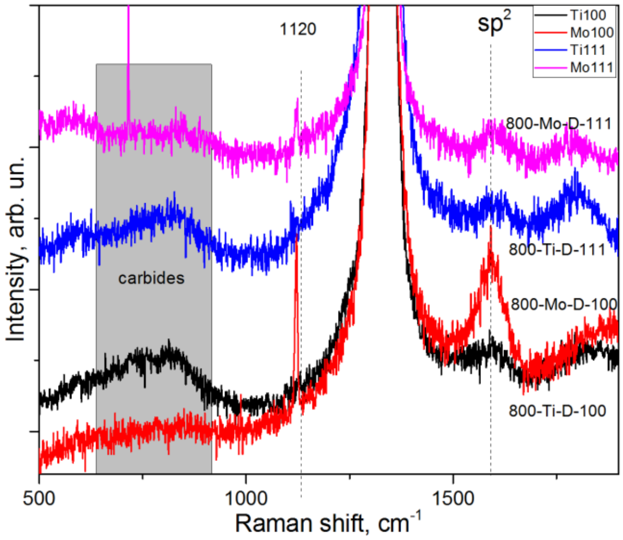

Figure S3. Raman spectra of metal-coated diamond substrates annealed at 800 ^{0}C normalized at the noise level**.**

4. Estimation of thicknesses of layers of sputtered metals and sp^2 carbon from XPS data

The thickness of the initial molybdenum and titanium films deposited on the diamond substrates was estimated from XPS data using the Thickogram method [1]. Decomposition of the Ti 2p (Mo 3d) XPS spectra showed oxidation of titanium to TiO_2 (molybdenum to MoO_3). Using the NIST Electron Effective Attenuation Length Database, the attenuation lengths of photoelectrons for TiO_2 or MoO_3 were calculated (error ± 10%) [2]. The obtained thicknesses of the titanium films in the Ti-D-111 and Ti-D-100 samples are about 0.2 and 0.3 nm, respectively. The thicknesses for the Mo-D-111 and Mo-D-100 samples are about 0.7 and 1.1 nm, respectively.

The thickness of the sp^2 carbon layer formed in the 800-Mo-D-100 sample above Mo_2C particles was estimated from the ratio of the Mo 3d spectra of the 500-Mo-D-100 and 800-Mo-D-100 samples. We used the equation $I=I_0 \cdot e^{(-d/\lambda)}$. Here λ is the attenuation lengths of photoelectrons in graphite, d is the thickness of the sp^2 carbon layer, I and I_0 are integrated intensities of the Mo 3d XPS spectra of the 800-Mo-D-100 and 500-Mo-D-100 samples, respectively. The attenuation length λ from graphite under Al Kα radiation is 2.4 nm. The calculation gives the thickness d of about 2.9 nm. It should be noted that this estimation assumed that the sp^2-hybridized carbon layer is uniform.

References

1. Cumpson, P.J. The Thickogram: A method for easy film thickness measurement in XPS. *Surf. Interface Anal.* **2000**, *29*, 403–406.
2. Powell, C.J.; Jablonski, A. *NIST Electron Effective-Attenuation-Length Database, Version 1.3, SRD 82*; National Institute of Standards and Technology: Gaithersburg, MD, USA, 2011.
